# Supplementary material for: 2-deoxyglucose transiently inhibits yeast AMPK signaling and triggers glucose transporter endocytosis, potentiating the drug toxicity
Source: PLoS Genet. 2022 Aug 11;18(8):e1010169. doi: 10.1371/journal.pgen.1010169 (PMC9398028; doi:10.1371/journal.pgen.1010169)
Supplement: S1 Table — (DOCX) [file pgen.1010169.s001.docx]

### SUPPLEMENTARY TABLE 1.

| **Name** | **Genotype & Description** | **Origin & Reference** |
| --- | --- | --- |
| ySL0066: **BY4741 (WT)** | *MAT a; ura3Δ0, his3Δ1, leu2Δ0, met15Δ0* | [1] |
| ySL0534: ***rod1Δ*** | *MAT a; ura3Δ0, his3Δ1, leu2Δ0, met15Δ0 rod1∆::KANMX* | Léon lab |
| ySL0541: ***reg1Δ*** | *MAT a; ura3Δ0, his3Δ1, leu2Δ0, met15Δ0 reg1∆::HIS3MX* | [2] |
| ySL0567: ***snf1Δ*** | *Mat alpha, ura3Δ0, his3Δ1, leu2Δ0, lys2∆0, snf1∆::kanMX4* | Léon lab |
| ySL0854: ***cnb1Δ*** | *MAT a,ura3Δ0, his3Δ1, leu2Δ0, met15Δ0 cnb1∆::KANMX* | Léon lab |
| ySL1027: **Hxt3-GFP** | *MAT a; ura3Δ0, his3Δ1, leu2Δ0, met15Δ0 HXT3::GFP-HIS3MX* | [3] |
| ySL1140: **Hxt2-GFP** | *MAT a;ura3Δ0, his3Δ1, leu2Δ0, met15Δ0 HXT2::GFP-HIS3MX* | [3] |
| ySL1186: **Hxt1-GFP** | *MAT a; ura3Δ0, his3Δ1, leu2Δ0, met15Δ0 HXT1::GFP-HIS3MX* | [3] |
| ySL1690: ***hxk2Δ*** | *MAT a; ura3Δ0, his3Δ1, leu2Δ0, met15Δ0 hxk2∆::KANMX* | Léon lab |
| ySL1852 : **Hxt4-GFP** | *MAT a; ura3Δ0, his3Δ1, leu2Δ0, met15Δ0 HXT4::GFP-HIS3MX* | [3] |
| ySL1961: ***slt2Δ*** | *MAT a; ura3Δ0, his3Δ1, leu2Δ0, met15Δ0 slt2∆::KANMX* | Euroscarf |
| ySL2092: **Vht1-GFP** | *MAT a; ura3Δ0, his3Δ1, leu2Δ0, met15Δ0 VHT1::GFP-HIS3MX* | [4] |
| ySL2110: **Pdr12-GFP** | *MAT a; ura3Δ0, his3Δ1, leu2Δ0, met15Δ0 PDR12::GFP-HIS3MX* | [4] |
| ySL2119: **Bap2-GFP** | *MAT a; ura3Δ0, his3Δ1, leu2Δ0, met15Δ0 BAP2::GFP-HIS3MX* | [4] |
| ySL2123: **Fps1-GFP** | *MAT a; ura3Δ0, his3Δ1, leu2Δ0, met15Δ0 FPS1::GFP-HIS3MX* | [4] |
| ySL2132: **Mid2-GFP** | *MAT a; ura3Δ0, his3Δ1, leu2Δ0, met15Δ0 MID2::GFP-HIS3MX* | [4] |
| ySL2142: **Qdr3-GFP** | *MAT a; ura3Δ0, his3Δ1, leu2Δ0, met15Δ0 QDR3::GFP-HIS3MX* | [4] |
| ySL2169: **Slg1-GFP** | *MAT a; ura3Δ0, his3Δ1, leu2Δ0, met15Δ0 SLG1::GFP-HIS3MX* | [4] |
| ySL2175: **Ste2-GFP** | *MAT a; ura3Δ0, his3Δ1, leu2Δ0, met15Δ0 STE2::GFP-HIS3MX* | [4] |
| ySL2204: ***hac1Δ*** | *MAT a; ura3Δ0, his3Δ1, leu2Δ0, met15Δ0 hac1∆::KANMX* | Euroscarf |
| ySL2216: **Itr1-GFP *snf1Δ*** | *MAT a; ura3Δ0, his3Δ1, leu2Δ0, met15Δ0 snf1∆::HPHNT1 Itr1::GFP-HIS3MX* | This study |
| ySL2219: **Tat1-GFP** | *MAT a; ura3Δ0, his3Δ1, leu2Δ0, met15Δ0 TAT1::GFP-HIS3MX* | [4] |
| ySL2221: **Can1-GFP** | *MAT a; ura3Δ0, his3Δ1, leu2Δ0, met15Δ0 CAN1::GFP-HIS3MX* | [4] |
| ySL2222: **Lyp1-GFP** | *MAT a; ura3Δ0, his3Δ1, leu2Δ0, met15Δ0 LYP1::GFP-HIS3MX* | [4] |
| ySL2223: **Fui1-GFP** | *MAT a; ura3Δ0, his3Δ1, leu2Δ0, met15Δ0 FUI1::GFP-HIS3MX* | [4] |
| ySL2224: **Itr1-GFP** | *MAT a; ura3Δ0, his3Δ1, leu2Δ0, met15Δ0 ITR1::GFP-HIS3MX* | [4] |
| ySL2237: **Pdr12-GFP *snf1Δ*** | *MAT a; ura3Δ0, his3Δ1, leu2Δ0, met15Δ0 PDR12::GFP-HIS3MX snf1∆::NATNT2* | This study |
| ySL2288: **Ina1-GFP** | *MAT a; ura3Δ0, his3Δ1, leu2Δ0, met15Δ0 INA1::GFP-HIS3MX* | [4] |
| ySL2315: ***hog1Δ*** | *MAT a; ura3Δ0, his3Δ1, leu2Δ0, met15Δ0 hog1∆::KANMX* | Euroscarf |
| ySL2325: **Ina1-GFP *npi1*** | *MAT a; ura3Δ0, his3Δ1, leu2Δ0, met15Δ0 INA1::GFP-HIS3MX pRSP5::KANMX* | This study |
| ySL2395: **Ina1-GFP** ***snf1Δ*** | *MAT a; ura3Δ0, his3Δ1, leu2Δ0, met15Δ0   snf1∆::HIS3MX INA1::GFP-HPHNT1* | This study |
| ySL2406: **Ina1-GFP *rod1Δ*** | *MAT a; ura3Δ0, his3Δ1, leu2Δ0, met15Δ0 INA1::GFP-HPHNT1 rod1∆::KANMX* | This study |
| ySL2412: **Ina1-GFP *rvs167Δ*** | *MAT a; ura3Δ0, his3Δ1, leu2Δ0, met15Δ0 rvs167∆KANMX4  INA1::GFP-HPHNT1* | This study |
| ySL2434: **Ina1-Δ5-GFP** | *MAT a; ura3Δ0, his3Δ1, leu2Δ0, met15Δ0 Ina1-∆5::GFP-KANMX* | This study |
| ySL2443: **Pil1-GFP** | *MAT a; ura3Δ0, his3Δ1, leu2Δ0, met15Δ0 PIL1::GFP-HIS3MX* | [4] |
| ySL2444: **Sur7-GFP** | *MAT a; ura3Δ0, his3Δ1, leu2Δ0, met15Δ0 SUR7::GFP-HIS3MX* | [4] |
| ySL2445: **Lsp1-GFP** | *MAT a; ura3Δ0, his3Δ1, leu2Δ0, met15Δ0   LSP1::GFP-HIS3MX* | [4] |
| ySL2446: **Seg1-GFP** | *MAT a; ura3Δ0, his3Δ1, leu2Δ0, met15Δ0 SEG1::GFP-HIS3MX* | [4] |
| ySL2451: **Lyp1-GFP *rod1Δ*** | *MAT a; ura3Δ0, his3Δ1, leu2Δ0, met15Δ0 LYP1::GFP-HIS3MX rod1∆::KANMX* | This study |
| ySL2452: **Qdr3-GFP *rod1Δ*** | *MAT a; ura3Δ0, his3Δ1, leu2Δ0, met15Δ0 QDR3::GFP-HIS3MX rod1∆::KANMX* | This study |
| ySL2545: **Tat1-GFP *rod1Δ*** | *MAT a; ura3Δ0, his3Δ1, leu2Δ0, met15Δ0 TAT1::GFP-HIS3MX rod1∆::KANMX* | This study |
| ySL2546: **Fui1-GFP *rod1Δ*** | *MAT a; ura3Δ0, his3Δ1, leu2Δ0, met15Δ0 FUI1::GFP-HIS3MX rod1∆::KANMX* | This study |
| ySL2580: **Vht1-GFP *rod1Δ*** | *MAT a; ura3Δ0, his3Δ1, leu2Δ0, met15Δ0 VHT1::GFP-HIS3MX rod1∆::URA3* | This study |
| ySL2591: **Ste2-GFP *rod1Δ*** | *MAT a; ura3Δ0, his3Δ1, leu2Δ0, met15Δ0 STE2::GFP-HIS3MX rod1∆::URA3* | This study |
| ySL2592: **Can1-GFP *rod1Δ*** | *MAT a; ura3Δ0, his3Δ1, leu2Δ0, met15Δ0 CAN1::GFP-HIS3MX rod1∆::URA3* | This study |
| ySL2623: **Wsc3-GFP** | *MAT a; ura3Δ0, his3Δ1, leu2Δ0, met15Δ0 WSC3::GFP-HIS3MX* | [4] |
| ySL2628: **Wsc2-GFP** | *MAT a; ura3Δ0, his3Δ1, leu2Δ0, met15Δ0 WSC2::GFP-HIS3MX* | [4] |
| ySL2671: **Ina1-GFP *hxk2Δ*** | *MAT a; ura3Δ0, his3Δ1, leu2Δ0, met15Δ0 ; INA1::GFP-HIS3MX  hxk2∆::KANMX* | This study |
| ySL2724: **Hxt1-GFP *rod1Δ*** | *MAT a; ura3Δ0, his3Δ1, leu2Δ0, met15Δ0   HXT1::GFP-HIS3MX rod1∆::HPHNT1* | This study |
| ySL2725: **Hxt3-GFP *rod1Δ*** | *MAT a; ura3Δ0, his3Δ1, leu2Δ0, met15Δ0   HXT3::GFP-HIS3MX rod1∆::HPHNT1* | This study |
| ySL2726: **Itr1-GFP *rod1Δ*** | *MAT a; ura3Δ0, his3Δ1, leu2Δ0, met15Δ0 ITR1::GFP-HIS3MX rod1∆::HPHNT1* | This study |
| ySL2727: **Pdr12-GFP *rod1Δ*** | *MAT a; ura3Δ0, his3Δ1, leu2Δ0, met15Δ0 PDR12::GFP-HIS3MX rod1∆::HPHNT1* | This study |
| ySL2728: **Lyp1-GFP *rod1Δ rog3Δ*** | *MAT a; ura3Δ0, his3Δ1, leu2Δ0, met15Δ0 LYP1::GFP-HIS3MX rod1∆::KANMX; rog3∆::NATNT2* | This study |
| ySL2739: **Vph1-mCherry** | *MAT a; ura3Δ0, his3Δ1, leu2Δ0, met15Δ0 ; Vph1-mCherry::NATNT2* | This study |
| ySL2746: ***rod1∆ hxt6∆*** | *MAT a; ura3Δ0, his3Δ1, leu2Δ0, met15Δ0 rod1∆::KANMX hxt6∆::NATNT2* | This study |
| ySL2880: **Ina1-GFP Pil1-mCherry** | *MAT a; ura3Δ0, his3Δ1, leu2Δ0, met15Δ0 INA1::GFP-HIS3MX PIL1::mCherry-NATNT2* | This study |
| ySL2965: *reg1****Δ*** | *MAT a; ura3Δ0, his3Δ1, leu2Δ0, met15Δ0 reg1∆::HPHNT2* | This study |
| ySL3003: ***rod1Δ* Hxt3-GFP** | *MAT a; ura3Δ0, his3Δ1, leu2Δ0, met15Δ0 rod1∆::KANMX Hxt3::GFP-HPHNT1* | This study |
| ySL3026: ***hxt3Δ*** | *MAT a; ura3Δ0, his3Δ1, leu2Δ0, met15Δ0 hxt3∆::KANMX* | Euroscarf |
| ySL3027: ***rod1Δhxt3Δ*** | *MAT a; ura3Δ0, his3Δ1, leu2Δ0, met15Δ0 hxt3∆::KANMX rod1∆::HPHNT1* | This study |
| ySL3028: ***hxt1Δ*** | *MAT a; ura3Δ0, his3Δ1, leu2Δ0, met15Δ0 hxt1∆::KANMX* | Euroscarf |
| ySL3029: ***rod1Δhxt1Δ*** | *MAT a; ura3Δ0, his3Δ1, leu2Δ0, met15Δ0 hxt1∆::KANMX rod1∆::HPHNT1* | This study |
| ySL3084: ***hxt1Δhxt3∆*** | *MAT a; ura3Δ0, his3Δ1, leu2Δ0, met15Δ0 hxt1∆::KANMX hxt3∆::HPHNT1* | This study |
| ySL3099: ***rod1∆ dog1∆ dog2∆*** | *MAT a; ura3Δ0, his3Δ1, leu2Δ0, met15Δ0 rod1∆::KANMX dog1∆dog2∆::LEU2* | This study |
| ySL3118 : **Hxt4-GFP *rod1∆*** | *MAT a; ura3Δ0, his3Δ1, leu2Δ0, met15Δ0 HXT4::GFP-HIS3MX rod1∆::KANMX* | This study |
| ySL3124: **Hxt2-GFP *rod1Δ*** | *MAT a; ura3Δ0, his3Δ1, leu2Δ0, met15Δ0 HXT2::GFP-HIS3MX rod1∆::HPHNT1* | This study |
| ySL3171: **Snf1-GFP** | *MAT a; ura3Δ0, his3Δ1, leu2Δ0, met15Δ0 SNF1::GFP-KANMX* | This study |
| ySL3224: ***hxk2∆*** **Snf1-GFP** | *MAT a; ura3Δ0, his3Δ1, leu2Δ0, met15Δ0 SNF1::GFP-KANMX hxk2∆::HIS3MX* | This study |
| ySL3226: ***hxk2∆*** **Hxt1-GFP** | *MAT a; ura3Δ0, his3Δ1, leu2Δ0, met15Δ0 HXT1::GFP-HIS3 hxk2∆::KANMX* | This study |
| ySL3227: ***hxk2∆*** **Hxt3-GFP** | *MAT a; ura3Δ0, his3Δ1, leu2Δ0, met15Δ0 HXT3::GFP-HIS3 hxk2∆::KANMX* | This study |
| ySL3231: ***reg1∆*** **Snf1-GFP** | *MAT a; ura3Δ0, his3Δ1, leu2Δ0, met15Δ0 SNF1::GFP-KANMX reg1∆::LEU2* | This study |

###

### References.

1. Brachmann CB, Davies A, Cost GJ, Caputo E, Li J, Hieter P, et al. Designer deletion strains derived from Saccharomyces cerevisiae S288C: a useful set of strains and plasmids for PCR-mediated gene disruption and other applications. Yeast. 1998;14(2):115-32. Epub 1998/03/04. doi: 10.1002/(SICI)1097-0061(19980130)14:2<115::AID-YEA204>3.0.CO;2-2. PubMed PMID: 9483801.

2. Becuwe M, Vieira N, Lara D, Gomes-Rezende J, Soares-Cunha C, Casal M, et al. A molecular switch on an arrestin-like protein relays glucose signaling to transporter endocytosis. J Cell Biol. 2012;196(2):247-59. Epub 2012/01/18. doi: 10.1083/jcb.201109113. PubMed PMID: 22249293.

3. Hovsepian J, Defenouillere Q, Albanese V, Vachova L, Garcia C, Palkova Z, et al. Multilevel regulation of an alpha-arrestin by glucose depletion controls hexose transporter endocytosis. J Cell Biol. 2017;216(6):1811-31. Epub 2017/05/05. doi: 10.1083/jcb.201610094. PubMed PMID: 28468835; PubMed Central PMCID: PMCPMC5461024.

4. Huh WK, Falvo JV, Gerke LC, Carroll AS, Howson RW, Weissman JS, et al. Global analysis of protein localization in budding yeast. Nature. 2003;425(6959):686-91. PubMed PMID: 14562095.
