# Supplementary material for: 2-deoxyglucose transiently inhibits yeast AMPK signaling and triggers glucose transporter endocytosis, potentiating the drug toxicity
Source: PLoS Genet. 2022 Aug 11;18(8):e1010169. doi: 10.1371/journal.pgen.1010169 (PMC9398028; doi:10.1371/journal.pgen.1010169)
Supplement: S2 Table — (DOCX) [file pgen.1010169.s002.docx]

### SUPPLEMENTARY TABLE 2.

Plasmids used in this study.

| **Name** | **Description** | **Origin & Reference** |
| --- | --- | --- |
| **pSL093** | pRS416-derived (CEN, *URA3*) *p_ROD1_:ROD1*-GFP | [1] |
| **pSL094** | pRS415-derived (CEN, *LEU2*) *p_ROD1_:ROD1*-3HA | [2] |
| **pSL205** | p*_CUP1_*:6xHis-Ub (2µ, *LEU2*) (pJD421) | [3] |
| **pSL237** | pRS316-derived (CEN, *URA3*) p_Rod1_:Rod1-3Flag | Olivier Vincent |
| **pSL409** | Yep358-based (2μ, *URA3) p_DOG1_(1000bp):LacZ* | [4] |
| **pSL410** | Yep358-based (2μ, *URA3) p_DOG2_(1000bp):LacZ* | [4] |
| **pSL412** | pRS426-derived (2μ, *URA3*) *p_GPD_:DOG2* | [4] |
| **pSL436** | pRS426-derived (2μ, *URA3*) *p_GPD_:DOG2(DD>AA)* | [4] |
| **pSL559** | pRS313-derived (CEN, *HIS3*) *p_ROD1_:ROD1*-Flag | This study |
| **pSL560** | pRS313-derived (CEN, *HIS3*) *p_ROD1_:ROD1-PYm*-Flag | This study |
| **pSL561** | pRS313-derived (CEN, *HIS3*) *p_ROD1_:ROD1-S12A*-Flag | This study |
| **pSL563** | pRS313-derived (CEN, *HIS3*) *p_ROD1_:ROD1-KR*-Flag | This study |
| **pSL589** | pUG35-derived (ARS/CEN, *URA3*) *pHXT3:HXT3*-GFP | This study |
| **pSL590** | pDRf1GW-ura3-derived (2µ, *URA3*) FLII12Pglu-700μδ6 Glucose FRET sensor, Addgene #28002 | Wolf Frommer [5] |
| **pSL591** | pUG35-derived (ARS/CEN, *URA3*) *pHXT3:HXT3(N370A)*-GFP | This study |
| **pSL599** | pRS426-derived (2μ, *URA3*) *p_HXT1_*:*HXT1* | This study |
| **pSL602** | pRS426-derived (2μ, *URA3*) *p_HXT3_*:*HXT3* | This study |
| **pSL608** | pDRF1-GW-derived (2µ, *URA3*), yAT1.03 ATP FRET sensor, Addgene #132781 | Bas Teusink [6] |
| **pSL609** | pDRF1-GW-derived (2µ, *URA3*), yAT1.03 ATP FRET sensor, mutated R122K,R126K (non-ATP binding), Addgene #132782 | Bas Teusink [6] |

**References.**

1. Becuwe M, Leon S. Integrated control of transporter endocytosis and recycling by the arrestin-related protein Rod1 and the ubiquitin ligase Rsp5. eLife. 2014;3:03307. doi: 10.7554/eLife.03307. PubMed PMID: 25380227; PubMed Central PMCID: PMC4244573.

2. Becuwe M, Vieira N, Lara D, Gomes-Rezende J, Soares-Cunha C, Casal M, et al. A molecular switch on an arrestin-like protein relays glucose signaling to transporter endocytosis. J Cell Biol. 2012;196(2):247-59. Epub 2012/01/18. doi: 10.1083/jcb.201109113. PubMed PMID: 22249293.

3. Dohmen RJ, Stappen R, McGrath JP, Forrova H, Kolarov J, Goffeau A, et al. An essential yeast gene encoding a homolog of ubiquitin-activating enzyme. J Biol Chem. 1995;270(30):18099-109. doi: 10.1074/jbc.270.30.18099. PubMed PMID: 7629121.

4. Defenouillėre Q, Verraes A, Laussel C, Friedrich A, Schacherer J, Leon S. The induction of HAD-like phosphatases by multiple signaling pathways confers resistance to the metabolic inhibitor 2-deoxyglucose. Sci Signal. 2019;12(597):aaw8000. doi: 10.1126/scisignal.aaw8000. PubMed PMID: 31481524.

5. Bermejo C, Haerizadeh F, Takanaga H, Chermak D, Frommer WB. Dynamic analysis of cytosolic glucose and ATP levels in yeast using optical sensors. Biochem J. 2010;432(2):399-406. doi: 10.1042/BJ20100946. PubMed PMID: 20854260; PubMed Central PMCID: PMC2992555.

6. Botman D, van Heerden JH, Teusink B. An Improved ATP FRET Sensor For Yeast Shows Heterogeneity During Nutrient Transitions. ACS Sens. 2020;5(3):814-22. doi: 10.1021/acssensors.9b02475. PubMed PMID: 32077276; PubMed Central PMCID: PMCPMC7106129.
