# Supplementary material for: 2-deoxyglucose transiently inhibits yeast AMPK signaling and triggers glucose transporter endocytosis, potentiating the drug toxicity
Source: PLoS Genet. 2022 Aug 11;18(8):e1010169. doi: 10.1371/journal.pgen.1010169 (PMC9398028; doi:10.1371/journal.pgen.1010169)
Supplement: S3 Table — (DOCX) [file pgen.1010169.s003.docx]

### SUPPLEMENTARY TABLE 3.

Antibodies used this study.

| **Name** | **Description** | **Dilution** | **Reference/Origin** |
| --- | --- | --- | --- |
| 𝛼 GFP | Mouse monoclonal against GFP, clones 7.1/ 13.1 | 1/5000 | 11814460001 -Roche |
| 𝛼 GFP | Goat polyclonal antibody against GFP (IRDye-800 conjugated, used to reveal Snf1-GFP) | 1/2000 | 600-132-215 -Rockland |
| 𝛼 Flag | Mouse monoclonal antibody against Flag | 1/5000 | F3165 - Sigma |
| 𝛼 pAMPK / pSnf1 | Rabbit polyclonal antibody against Thr172-phosphorylated human AMPKα | 1/1000 | #2535 – Cell Signaling Technology |
| 𝛼 polyHis tag | Mouse monoclonal antibody to reveal poly-His-tagged proteins incl. Snf1 (contains a stretch of 13 His residues) | 1/2000 | H1029 - Sigma |
| 𝛼 Ubiquitin | Mouse monoclonal antibody against ubiquitin | 1/1000 | P4D1 - Santa Cruz |
| 𝛼 Rabbit IgG | Goat secondary antibody against Rabbit IgG (HRP) | 1/5000 | A6154 - Sigma |
| 𝛼 Mouse IgG | Goat secondary antibody against Mouse IgG (HRP) | 1/5000 | A5278 - Sigma |
