# Supplementary figures and images for: 2-deoxyglucose transiently inhibits yeast AMPK signaling and triggers glucose transporter endocytosis, potentiating the drug toxicity
Source: PLoS Genet. 2022 Aug 11;18(8):e1010169. doi: 10.1371/journal.pgen.1010169 (PMC9398028; doi:10.1371/journal.pgen.1010169)

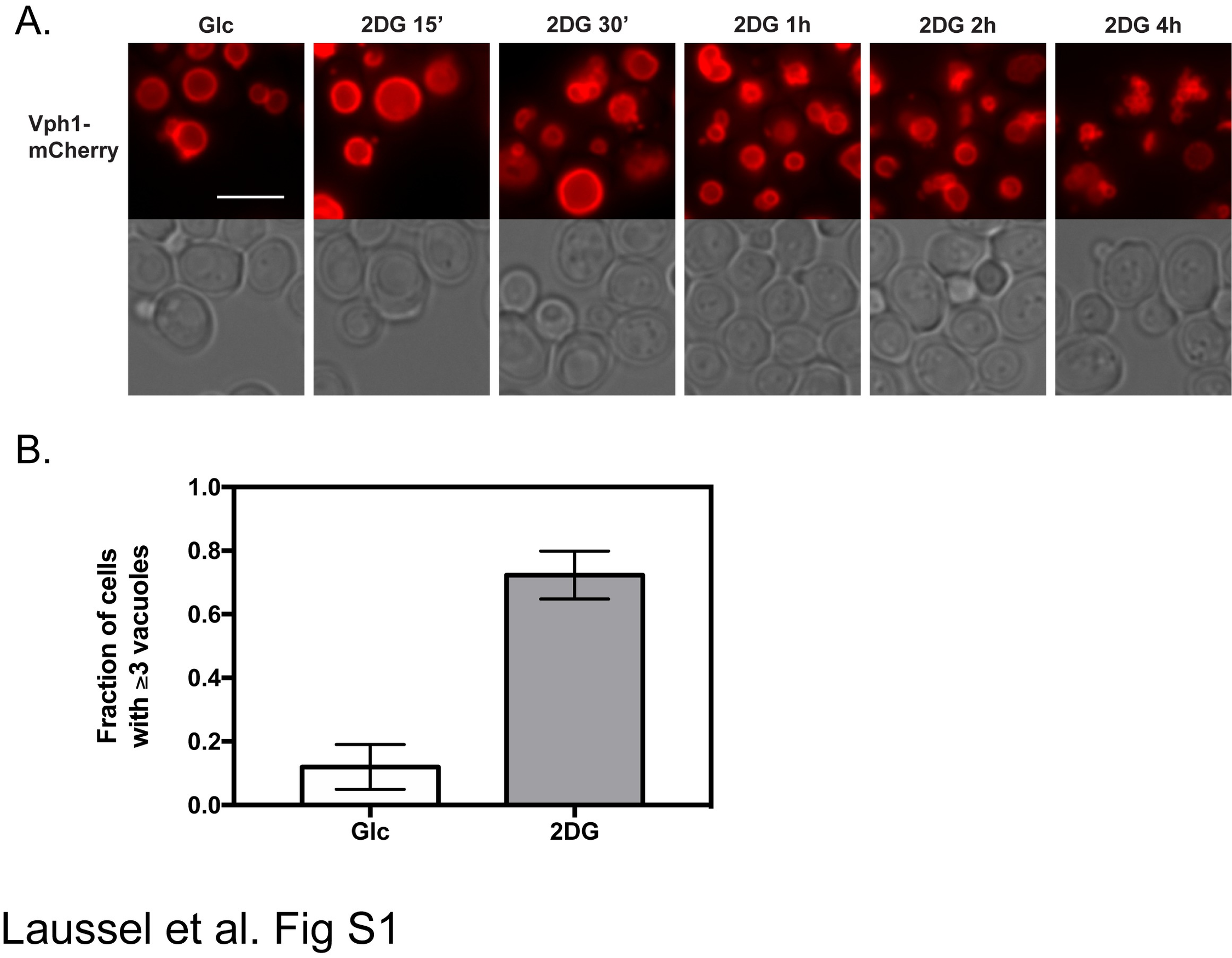

Supplement: S1 Fig — A. WT cells expressing Vph1 tagged with mCherry were grown overnight in glucose medium (exponential phase) and treated with 0.2% 2DG. Cells were collected and observed by fluorescence microscopy at the indicated times. Scale bar: 5 μm. B. Quantification of 2DG-induced vacuolar fragmentation (values ± SD, n = 3 independent experiments). (TIF) [file pgen.1010169.s004.tif]

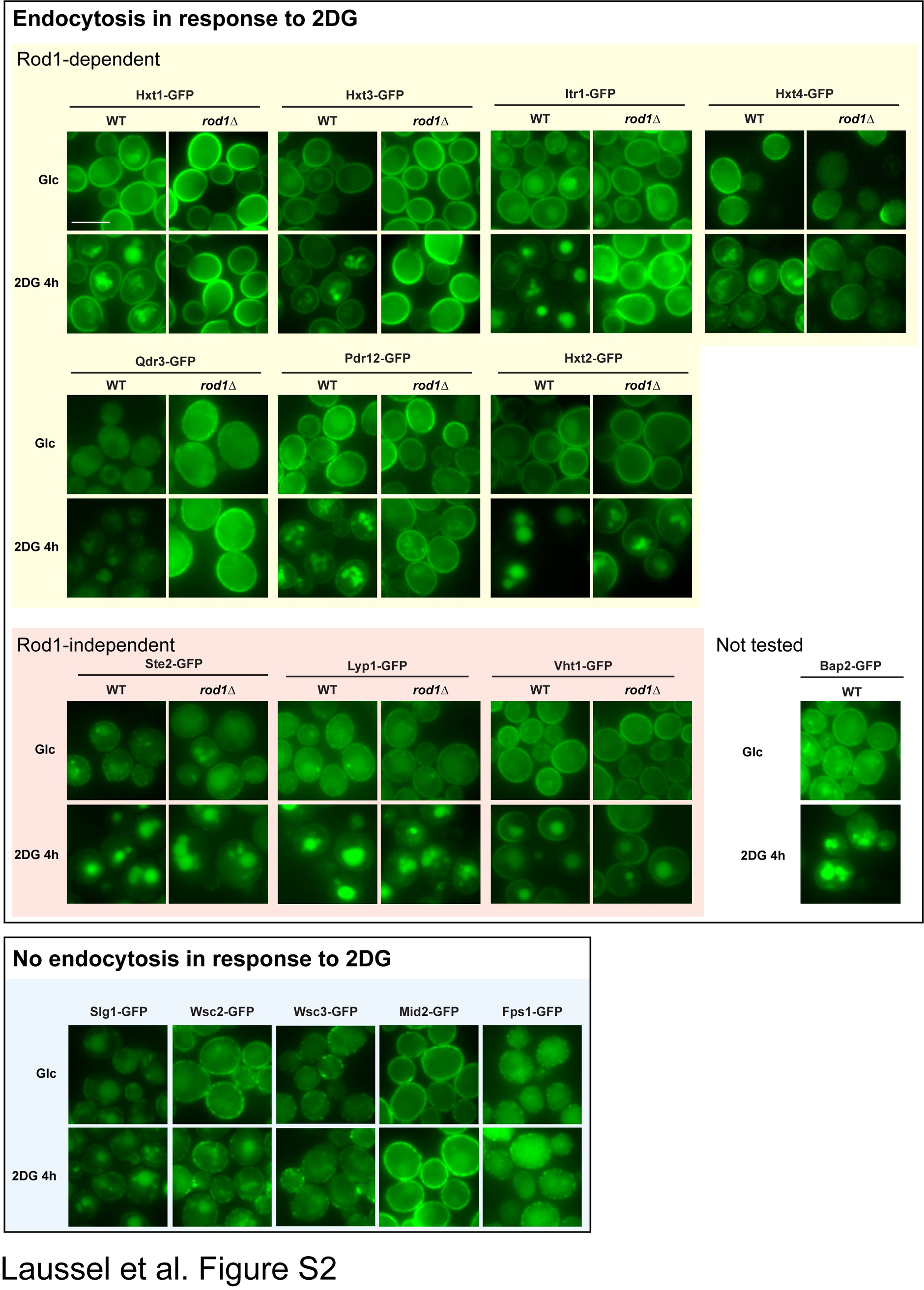

Supplement: S2 Fig — WT or rod1Δ cells expressing the indicated membrane proteins tagged with GFP at their endogenous genomic locus were observed by fluorescence microscopy before and after treatment with 2DG for 4h. Scale bar, 5 μm. (TIF) [file pgen.1010169.s005.tif]

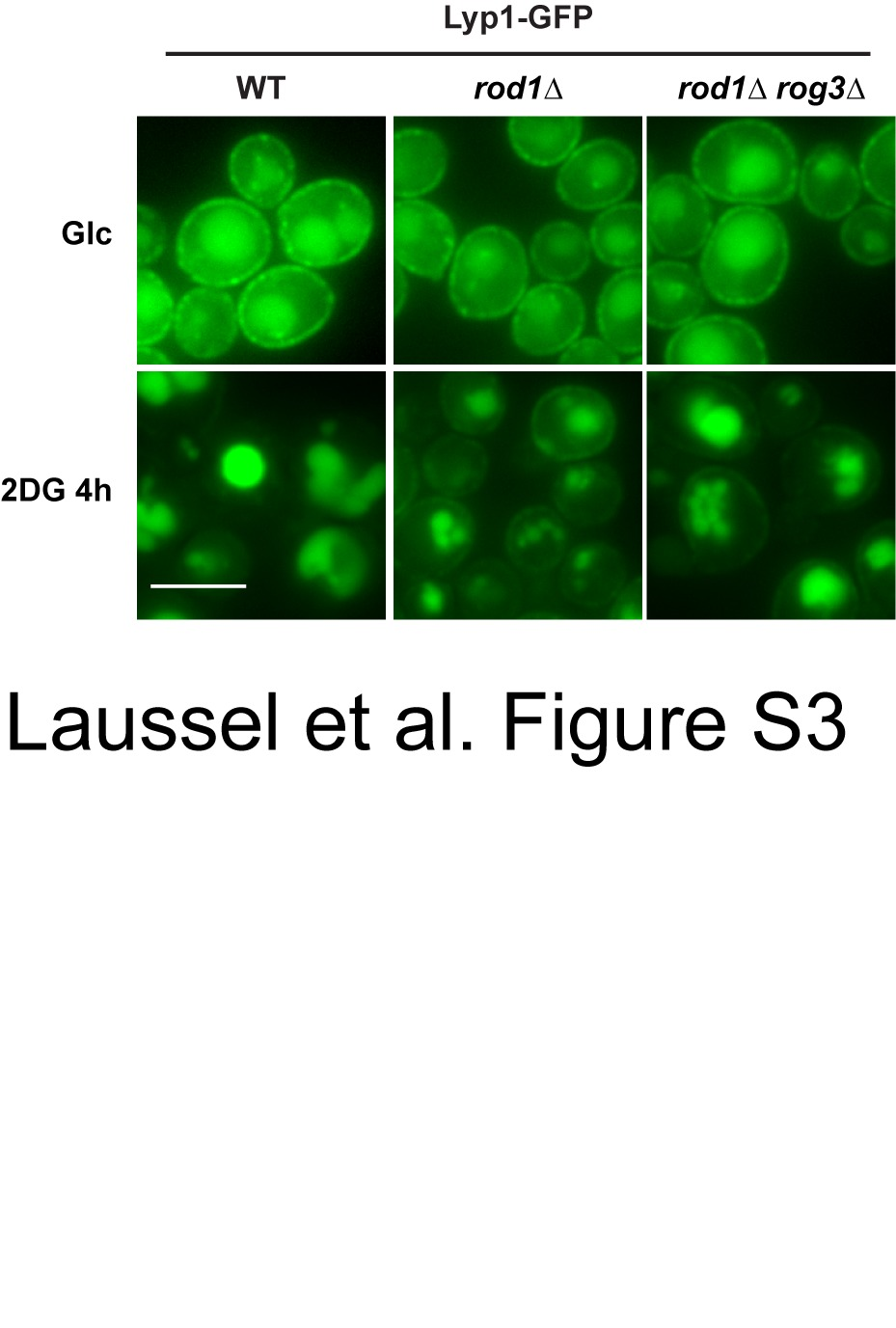

Supplement: S3 Fig — WT, rod1Δ and rod1Δ rog3Δ cells expressing Lyp1-GFP tagged at its endogenous genomic locus were observed by fluorescence microscopy before and after treatment with 2DG for 4h. Scale bar, 5 μm. (TIF) [file pgen.1010169.s006.tif]

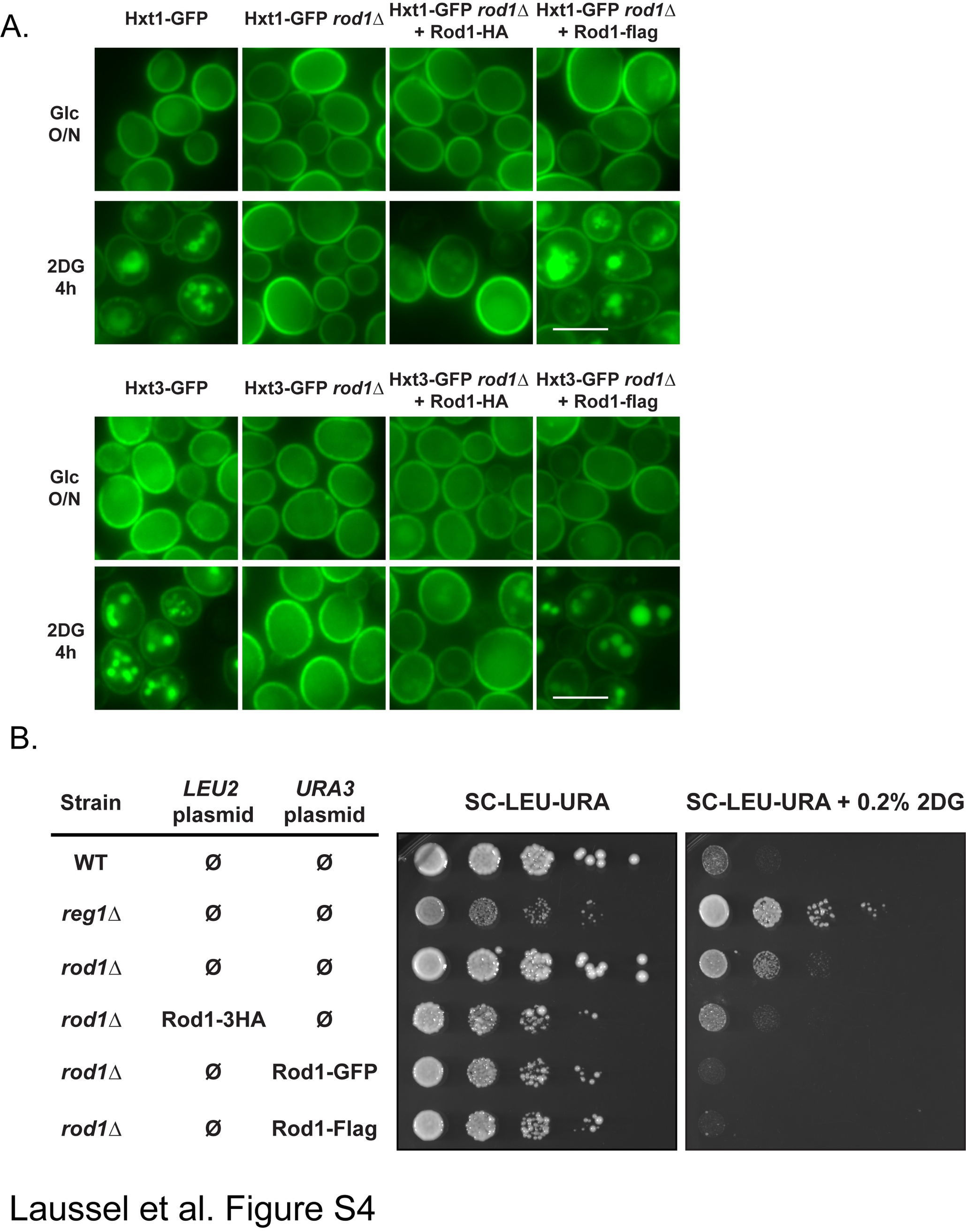

Supplement: S4 Fig — (A) The indicated strains were observed by fluorescence microscopy after growth in a glucose-containing medium and after 2DG treatment for 4h. Scale bar, 5 μm. (B) Serial dilutions of cultures of the indicated mutants/plasmid combinations were spotted on the indicated media and grown for 3 days at 30°C. (TIF) [file pgen.1010169.s007.tif]

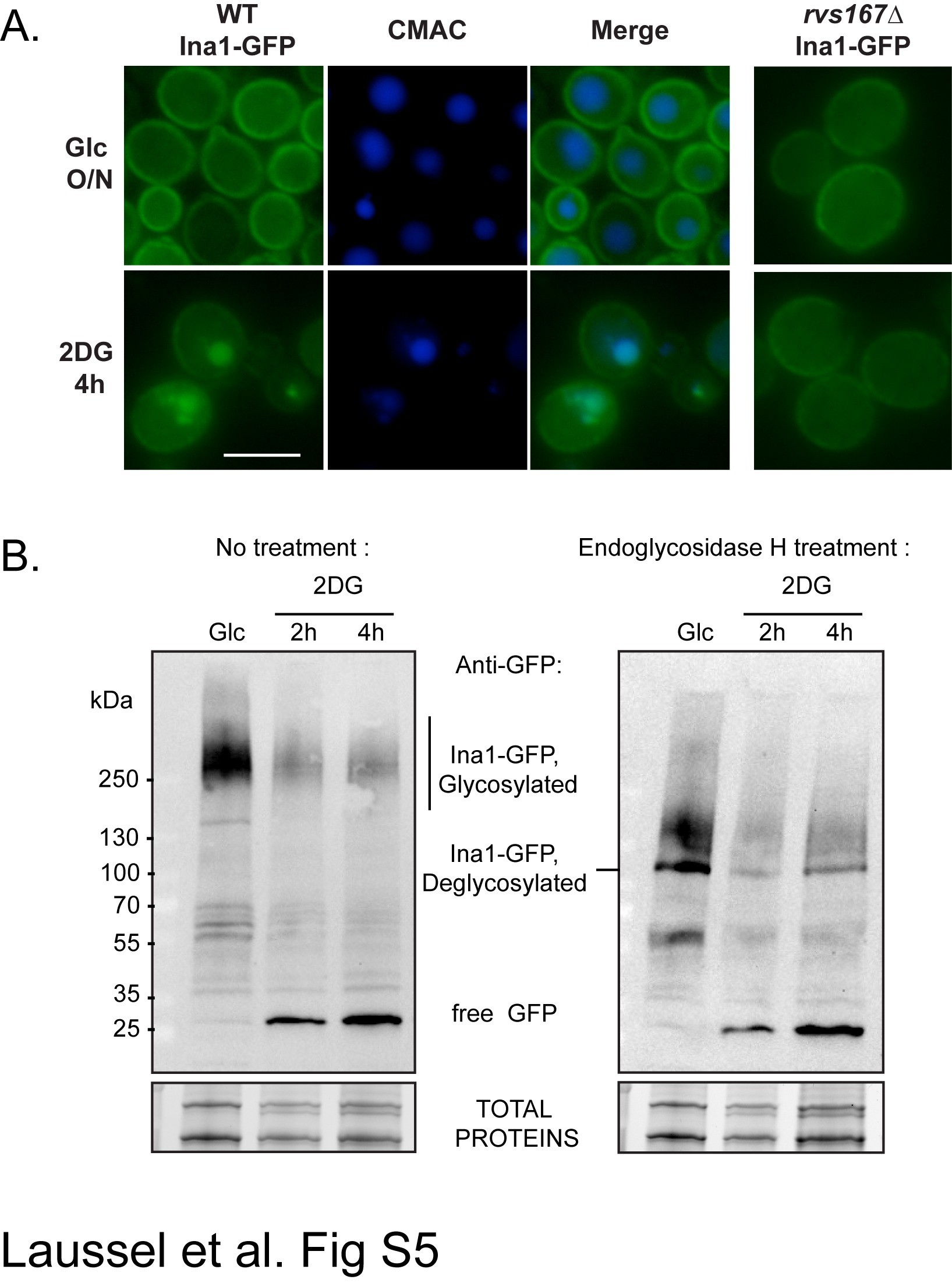

Supplement: S5 Fig — (A) WT or rvs167Δ strains expressing Ina1-GFP were observed by fluorescence microscopy after growth in a glucose-containing medium and after 2DG treatment for 4h. In addition, WT cells were incubated with CMAC to label the vacuole. Scale bar, 5 μm. (B) Left, Total protein extracts of WT cells expressing Ina1-GFP were prepared before and after 2h or 4h 2DG treatment and immunoblotted using anti-GFP antibodies. Right, the same extracts were treated with endoglycosidase H and immunoblotted using anti-GFP antibodies. (TIF) [file pgen.1010169.s008.tif]

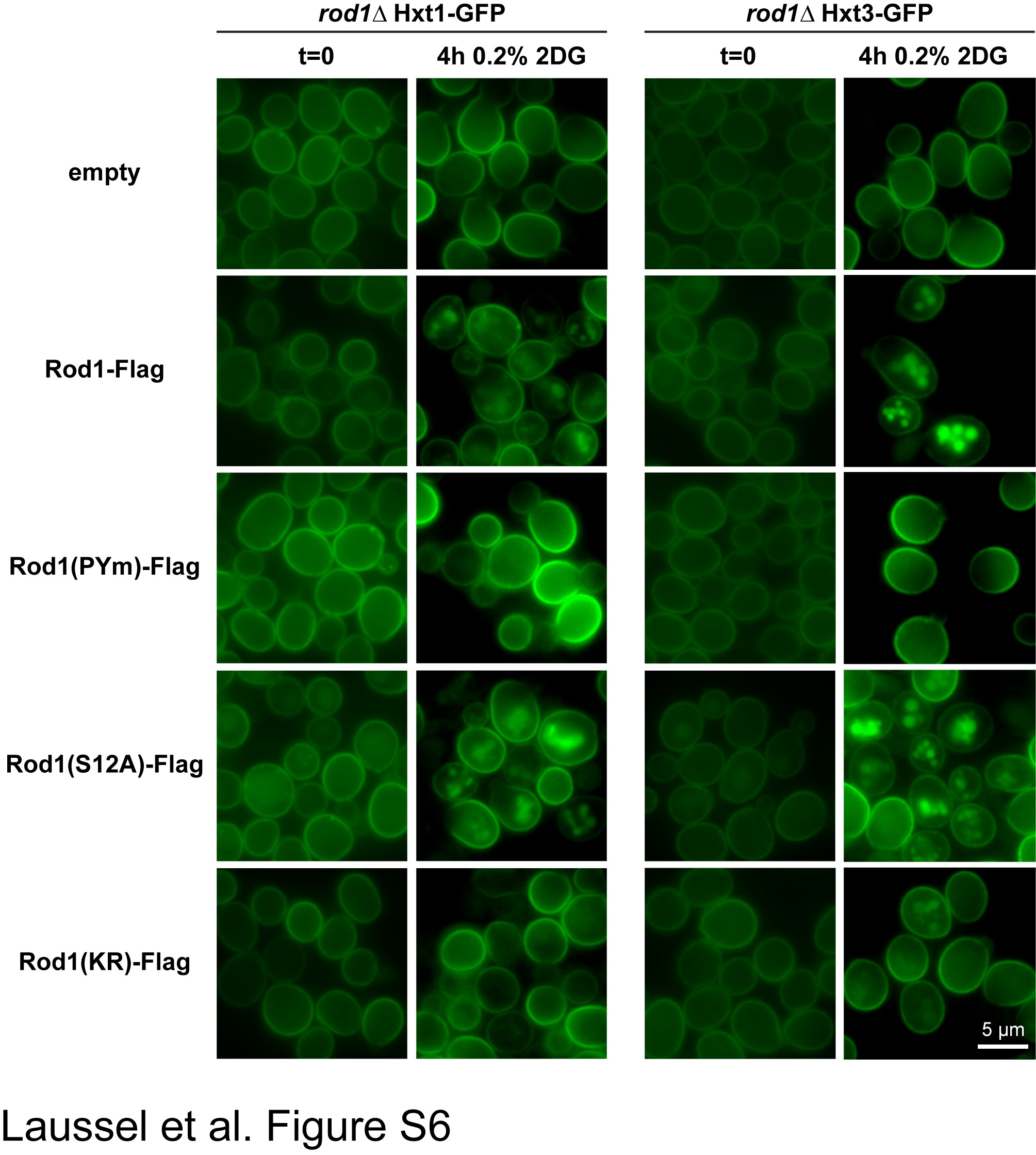

Supplement: S6 Fig — The indicated strains were observed by fluorescence microscopy after growth in a glucose-containing medium and after 2DG treatment for 4h. Scale bar, 5 μm. (TIF) [file pgen.1010169.s009.tif]

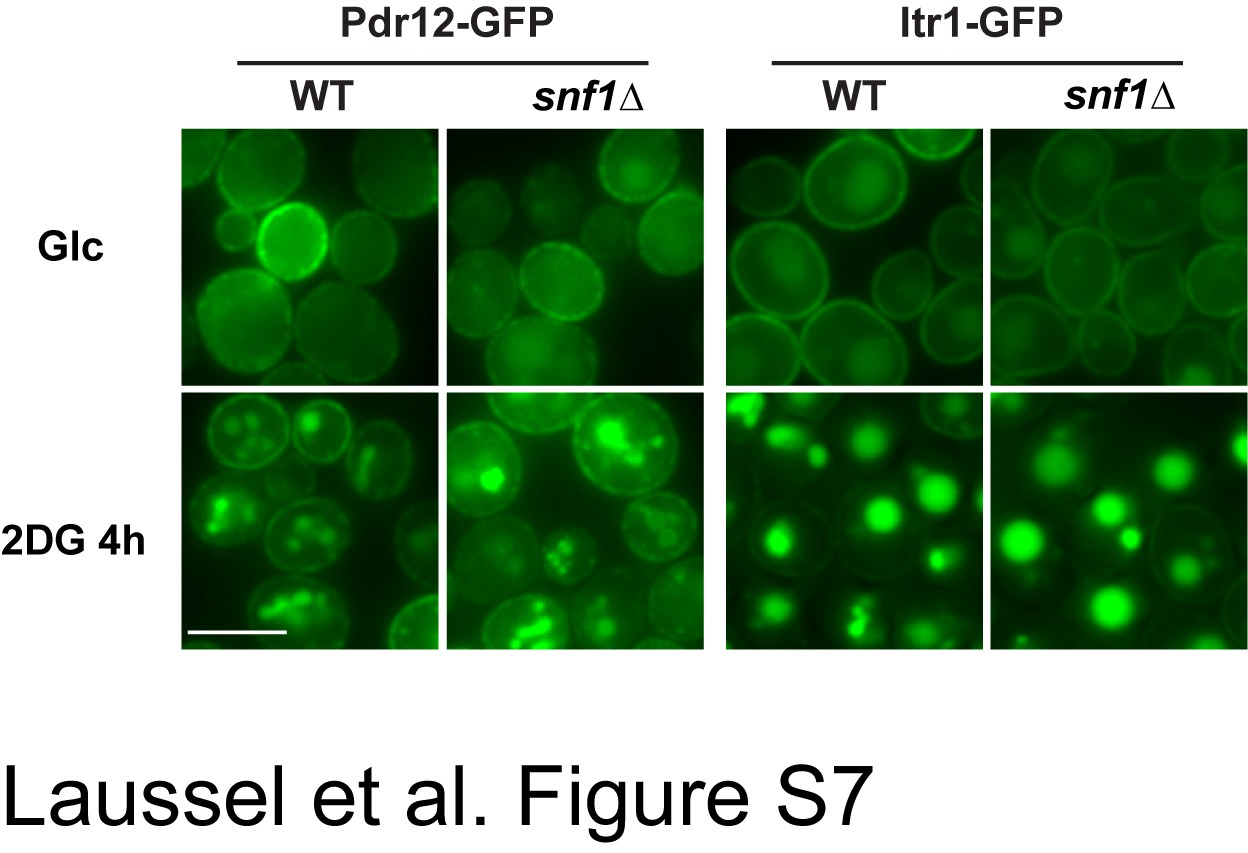

Supplement: S7 Fig — Pdr12-GFP, Pdr12-GFP snf1Δ, Itr1-GFP and Itr1-GFP snf1Δ cells were grown in a glucose-containing medium and observed by fluorescence microscopy before and after 2DG treatment for 4h. Scale bar, 5 μm. (TIF) [file pgen.1010169.s010.tif]

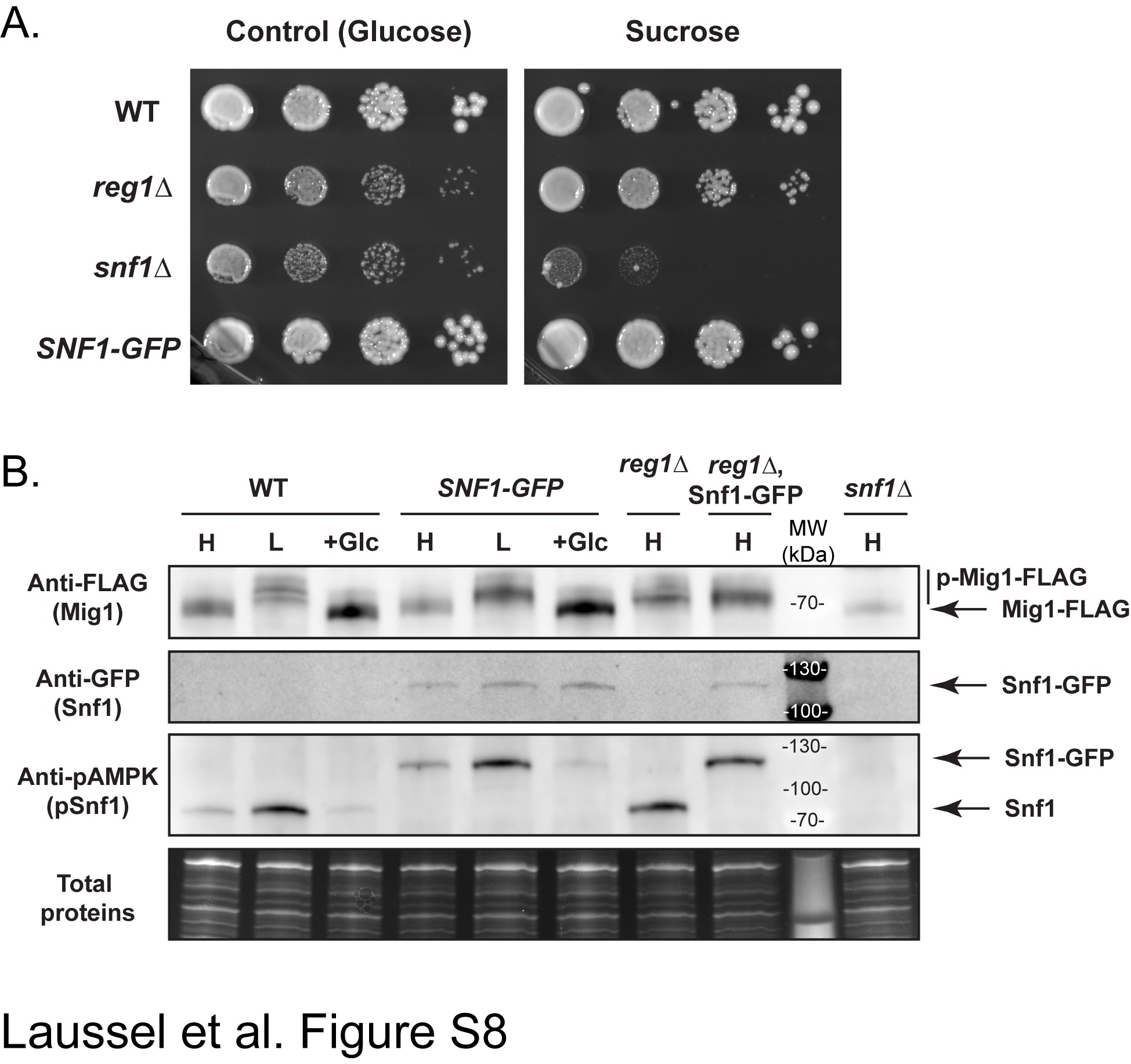

Supplement: S8 Fig — A. Serial dilutions of cultures of the WT, reg1Δ, snf1Δ and Snf1-GFP strains were spotted on synthetic complete medium containing either glucose or sucrose as a carbon source and grown for 3 days at 30°C. B. Total protein extracts of WT, Snf1-GFP, reg1Δ, reg1Δ Snf1-GFP and snf1Δ cells expressing Mig1-Flag were prepared after overnight growth (exponential phase) in glucose medium (H: high glucose), after transfer to 0.05% glucose for 2h (L: low glucose) and after addition of glucose for 10 min (+Glc). Samples were immunoblotted using anti-Flag, anti-GFP and anti-pAMPK antibodies.MW: molecular weight marker. (TIF) [file pgen.1010169.s011.tif]

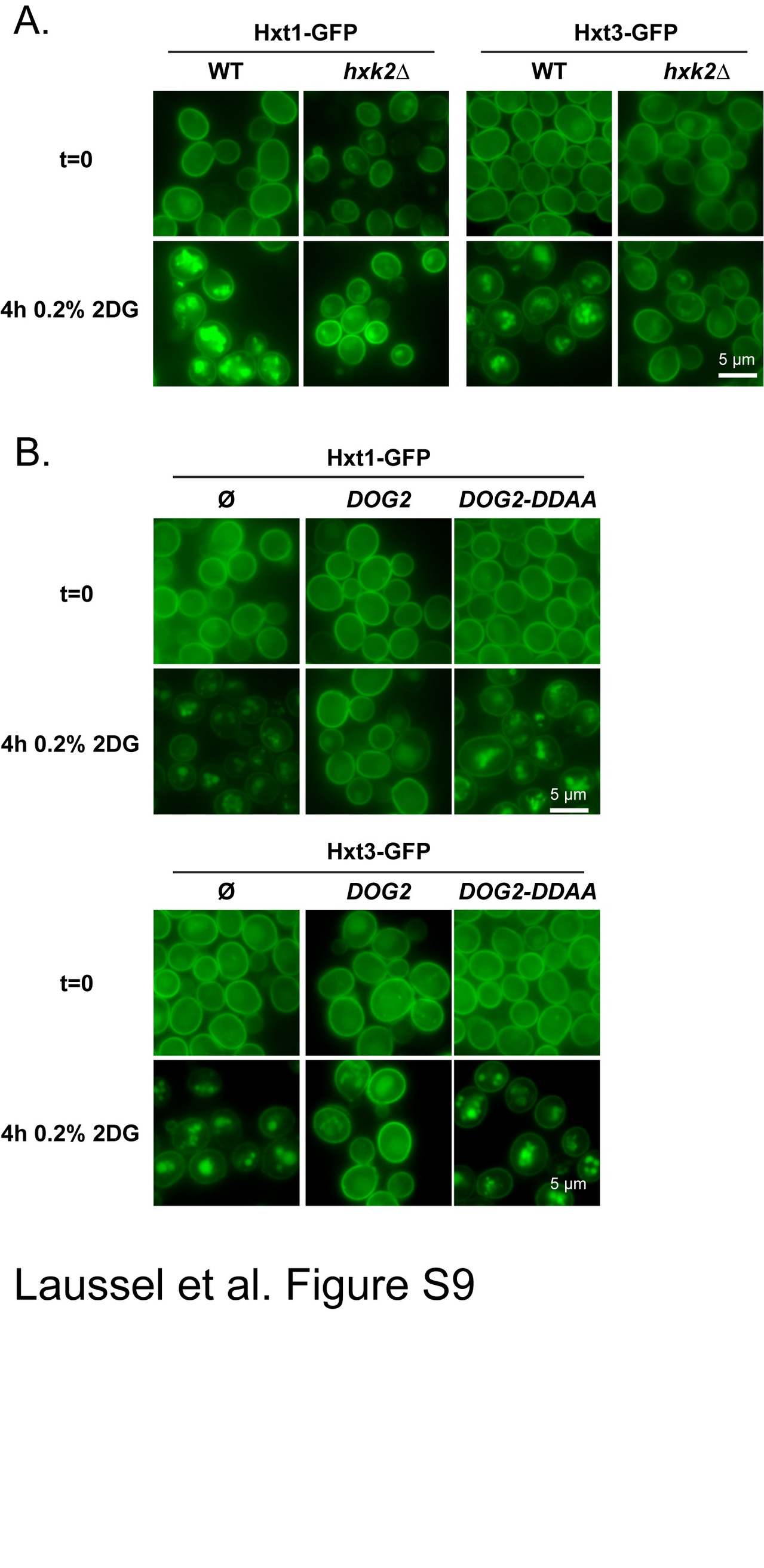

Supplement: S9 Fig — A. Hxt1-GFP, Hxt1-GFP hxk2Δ, Hxt3-GFP and Hxt3-GFP hxk2Δcells were grown in a glucose-containing medium and observed by fluorescence microscopy before and after 2DG treatment for 4h. Scale bar, 5 μm. B. Strains expressing Hxt1-GFP and Hxt3-GFP containing an empty plasmid (Ø) or plasmids allowing the overexpression of DOG2 or its catalytic mutant DOG2-DDAA were grown in a glucose-containing medium and observed by fluorescence microscopy before and after 2DG treatment for 4h. Scale bar, 5 μm. (TIF) [file pgen.1010169.s012.tif]
