# Supplementary material for: 2-deoxyglucose transiently inhibits yeast AMPK signaling and triggers glucose transporter endocytosis, potentiating the drug toxicity
Source: PLoS Genet. 2022 Aug 11;18(8):e1010169. doi: 10.1371/journal.pgen.1010169 (PMC9398028; doi:10.1371/journal.pgen.1010169)
Supplement: Attachment — Submitted filename: PLOS Genetics Review.pdf [file pgen.1010169.s013.pdf]

The paper entitled "2-deoxyglucose inhibits yeast AMPK signaling and triggers glucose transporter endocytosis, potentiating the drug toxicity" reports that 2DG regulates the endocytosis of many plasma membrane proteins, mostly in a Rod1-dependent manner. The data reported in this paper are interesting and they fit into a logical model. The quality of the work is high, indicating that the experiments were carried out with great care and precision. However, a number of results are rather confirmatory of previously published data (Refs. 16, 28, 31, 32).

Majors:

1. Because addition of glucose or 2DG to glucose-starved cells results in a sudden increase in PP1 activity, the authors state that: "Snf1 dephosphorylation by 2DG required the PP1 subunit Reg1, thus mimicking the effect that glucose has on glucose-starved cells (lines 213-214)." and that: "this mimicked the situation described when glucose-starved cells are treated with glucose (lines 217-218)." However, these statements are premature and lack adequate experimental evidence. Given that Rod1 is activated (ubiquitinated) in response to high glucose, the low affinity glucose transporters Hxt1 and Hxt3 would be primary targets of Rod1 (according to their claim) under such conditions. However, they are not endocytosed but are stable and remain functional when cells are exposed to high glucose levels. Although PP1 activity is increased in response to both high glucose and 2DG, the glucose transporters are endocytosed in response to 2DG but not to high glucose. The authors should better differentiate between high glucose-induced endocytosis (Jen1) and 2DG-induced endocytosis (Hxt1 and Hxt3) in glucose-grown cells.

2. 2DG-induced phosphorylation/activation of Snf1 is modest yet sufficient for the increased phosphorylation of Rod1 (Ref. 16). The authors observed a sharp but transient decrease in Snf1 phosphorylation shortly after 2DG addition. Their results show that Snf1 activity decreases immediately after 2DG treatment and increases within ½ - 1 hr after 2DG treatment (Figs. 3 B and 3C). However, the investigation of the functional significance of the 2DG-induced transient inactivation of Snf1 was not properly conducted. 2DG-induced endocytosis was not assessed by fluorescence microscopy until 4 hours after 2DG treatment. One can assume that Snf1 is active in cells treated with 2DG for more than an hour and regulates 2DG-indcued endocytosis.

3. The authors claim that Rod1-flag behaves functionally as the wild-type protein, because expression of Rod1-3HA does not restore endocytosis nor sensitivity to 2DG in a *rod1Δ* context, contrary to Rod1-flag (Fig. S4). However, expression of Rod1-3HA restores sensitivity (Fig. S4B) and endocytosis of Hxt1 (Ref. 32) and Jen1 (Ref. 28) in a *rod1Δ* context. Interestingly, the *rod1Δ* mutant is shown to be resistant to 2DG in figures 5B, 5C, and 6F but not in Fig. S4B upper panel, which might cause misinterpretation of the results. This should be clarified to avoid any confusion in the matter.

4. The lack of quantification of the plasma membrane and intracellular GFP fluorescence intensities measured in this manuscript does not allow a rigorous evaluation of protein turnover and recruitment. For example, vacuolar fragmentation is observed in 2DG-treated cells, but it is unclear whether Ina1-GFP is endocytosed in response to 2DG. 2DG (or some drugs) causes ER stress-induced vacuolar fragmentation (Fig. S1), which however does not directly reflect protein endocytosis. The results would have also been stronger if immunoblot analysis (of plasma membrane fractions) were provided to back up the fluorescence microscopy.

5. The abstract of the paper claims that: "Rod1 is central to 2DG-induced endocytosis because 2DG, following its phosphorylation by hexokinase Hxk2, triggers changes in Rod1 post-translational modifications and promotes its function in endocytosis." However, there are many PM proteins that are endocytosed in a 2DG--induced, Rod1-independent manner (Fig. S2). Furthermore, expression of a constitutively active (dephosphorylated/ubiquitinated) Rod1 (Rod1-S12A-Flag) does not lead to 2DG-independent endocytosis of Ina1-GFP (Fig. 2), suggesting that 2DG might have a limited role in translational modification of Rod1 (phosphorylation & ubiquitination). This sentence should be toned down.

Minors:

1. The title of the manuscript does not reflect its contents accurately. It is unclear whether 2DG inactivation of Snf1 is required for endocytosis, because the 2DG effect on Snf1 activity is transitory. In addition, the main focus of the paper is actually on Ina1 but not on glucose transporters. This should be reflected in the title.

2. The authors also investigated how *ROD1* deletion results in 2DG resistance and concluded that stabilization of Hxt1 and Hxt3 glucose transporters confers 2DG resistance to the *rod1Δ* mutant. These results are what would be expected, given that Rod1 removal increases the growth of 2DG-treated cells (Ref. 16, 32).
