# Supplementary material for: 2-deoxyglucose transiently inhibits yeast AMPK signaling and triggers glucose transporter endocytosis, potentiating the drug toxicity
Source: PLoS Genet. 2022 Aug 11;18(8):e1010169. doi: 10.1371/journal.pgen.1010169 (PMC9398028; doi:10.1371/journal.pgen.1010169)
Supplement: Attachment — Submitted filename: new Rebuttal.docx [file pgen.1010169.s014.docx]

**Reviewer #1:**

Clotilde Laussel and co-workers have used a combination of genetic, imaging and biochemical approaches to convincingly demonstrate that 2-deoxyglucose (2DG) inhibits AMPK signaling and thereby triggers a-arrestin (Rod1) mediated glucose transporter endocytosis in budding yeast, S.cerevisae. This process is detrimental for cells and potentiates the toxicity of 2DG.

On the mechanistic levels they show that the majority of 2DG enters the cells via the glucose transporters Hxt1 and Hxt3. The hexokinase Hxk2 rapidly converts 2DG into 2DG6P which then induces a transient activation of protein phosphatase 1 (PP1) and a concomitant inactivation of Snf1/AMPK. This results in the activation of the a-arrestin Rod1. Once activated, Rod1 enlists the HECT-type ubiquitin ligase Rsp5 to selectively downregulate a number of nutrient transporters, including the glucose transporters Hxt1 and Hxt3. The downregulation of Hxt1/3 exacerbates the metabolic blockade caused by 2DG (2DG6P) and ultimately leads to metabolic collapse and cell death. Interestingly loss of Rod1 causes 2DG tolerance. This might seem counterintuitive at first glance (more Hxt1/3 at the plasma membrane = more import of 2DG), however the authors have solved this conundrum.

They provide convincing evidence that in a rod1 mutant, Hxt1/3 are no longer downregulated in response to 2DG treatment. Surprisingly, this helps cells to detoxify 2DG, most likely by continuous import of glucose (resulting in metabolic competition) and by export of 2DG (or 2DG6P).

Overall, this is an interesting paper and should be published. The paper highlights the difference in AMPK signaling and 2DG action budding yeast and human cells, and it also offers a very interesting perspective on how a-arrestins dependent transporter regulation contributes to the mechanism of drug action.
We thank the reviewer for the time taken to assess our work and the constructive comments.

Comments (most of which are really minor):

1. Fig. S4b – Please directly compare the 2DG resistance of rod1 mutants + Rod1-Flag and rod1 mutants + Rod1-HA on the same agar plates.
This is now included as new Fig S4B. The results confirm our observations that Rod1-Flag (and Rod1-GFP) restore 2DG sensitivity to the *rod1∆* mutant but that the function of Rod1-HA is partially impaired.

3. Please specify the lysine residues in Rod1 that are ubiquitinated by Rsp5 (not just Rod1KR).
This is now specified in the Results section.

4. Along the same lines, please specify the 12 serine residues in Rod1 that are phosphorylated by Snf1.
This was mentioned in the method, so we added “see Material and methods” in the main text.

5. Fig. 2: Do mutations - Rod1KR, Rod1PYm and Rod1S12A also influence other Rod1 cargoes in addition to Ina1 (please also show for Hxt1/3).
This is now included as Fig S6. The results confirm our observations made on Ina1, i.e. the PYm and KR mutations are no longer able to restore the endocytosis of Hxt1 and Hxt3.

5. Fig. 4: Do mutations – hxk2 mutants and overexpression of Dog2 (and the mutant version) also influence other Rod1 cargoes in addition to Ina1 (please also show for Hxt1/3).
This is now included as Fig S9. Similarly to our observations on Ina1-GFP, Hxt1-GFP and Hxt3-GFP endocytosis are strongly impacted by HXK2 deletion and *DOG2* overexpression, except when the latter is mutated to be catalytically inactive.

6. Please check Snf1 phosphorylation at 4h 2DG treatment. Perhaps Snf1 only becomes hyperactive at later timepoints (4h)?
In the last few months, we've faced difficulties in visualizing Snf1 by immunoblots using the anti-His antibodies. The signal was already quite weak before (see Fig 4, A & B) but it has recently reached a point where we cannot do these experiments anymore. We tried another lot of the same antibodies (H1029, Sigma), other anti-His either published (Abcam ab1187; see Simpson-Lavy et al. 2017) or unpublished (H3, Santa Cruz ; H15, Santa Cruz ; 70796-3, Merck-Millipore) and even anti-hAMPK-alpha antibodies (D-6, Santa Cruz) with no success. For example, we could not provide a control for total Snf1 for panel 4D.
To get around this problem, and to avoid the use of a plasmid which could lead to Snf1 overexpression, we used a strain expressing Snf1-GFP tagged at its endogenous locus. This version of Snf1 was fully functional, as is now reported in Fig S8: (i) unlike a *snf1∆* strain, the Snf1-GFP strain grows like a wild-type on sucrose, (ii) Mig1-Flag phosphorylation was similar in the Snf1-GFP and the WT strains, (iii) Snf1-GFP phosphorylation increased at low glucose concentrations, which was counteracted by glucose addition, and finally (iv) Snf1-GFP phosphorylation increased in the *reg1∆* mutant. These data validate Snf1-GFP as a suitable epitope-tagged version of Snf1. This is now mentioned in the manuscript (l. 214-219).

The conditions to monitor Snf1 phosphorylation now being set up, we repeated the kinetics of Snf1 phosphorylation (original Fig 3C) and extended to 4h as requested. We also checked for Rod1 in the same experiment to answer to Reviewer 2, point 4. This is now included as a new Fig 3C. The results indicate that indeed, Snf1 is dephosphorylated in response to 2DG, and phosphorylation appears gradually over time to similar or even higher levels than before treatment. Similarly, Rod1 is dephosphorylated and seems gradually re-phosphorylated over time. These data clarify our views on the effects of 2DG on AMPK signaling in yeast. This is now mentioned in the Results section (l. 222-223) and the Discussion section (l. 370-372).

To be more clear and rigorous, we also changed the title of the article to add the word “transiently”: **2-deoxyglucose transiently inhibits yeast AMPK signaling and triggers glucose transporter endocytosis, potentiating the drug toxicity**

Note that we also seized the opportunity to repeat the experiment presented in panel 4D with Snf1-GFP, the results are the same but are better controlled since we now show total Snf1-GFP levels.

7. Fig. 5: I’m not sure that I fully can follow this argument: ‘Importantly, deletion of the gene encoding the amphiphysin orthologue RVS167, involved in endocytosis, did not result in 2DG resistance. Thus, it seems that only a partial endocytosis defect such as that observed in the rod1∆ (or rod1∆ rog3∆) mutant causes 2DG resistance.’ Could it be the rvs167 mutant is very sick anyways (in contrast to the rod1) and hence has trouble growing on 2DG? Also does loss of rvs167 really block Hxt1/3 endocytosis?
A comment was raised by reviewer 2 on the same aspect ("other comments" / point 9). Indeed, many of the "strong" endocytosis mutants are usually slow growers (eg. *vrp1∆*, *sla2∆*), making it difficult to conclude on their sensitivity/resistance to drugs. All in all, we agree with both reviewers that the argument is weak so we removed the corresponding panel and comment.

8. Figure 6B: The authors make a point that there is no difference in the intracellular 2DG levels between WT and rod1 mutants. I disagree – there is less 2DG in the rod1 mutant, but the difference is just not statistically significant. Also please do not use the * - *** system, just indicate the respective p-values (also for NS).
We rather think that the lack of statistical significance means that we don’t have sufficient confidence to be sure about a difference between the WT and *rod1* mutant (see Zieffler et al. (2019) "Statistical Thinking: A simulation approach to uncertainty"). Note that the same experiment (now presented in Panel 6D) leads to the same observation but again without statistical significance.

We indicated p-values on the graphs as suggested.

9. The authors argue that 2DG or 2DG6P is pumped out of the cells. Do they really provide evidence for that? Cloud they measure extracellular 2DG or 2DG6P measured (perhaps after chasing it?). If not please tone down the argument.
Thank you for the comment. Indeed, we only provide evidence of disappearance of 2DG6P from cells, which could be due to dephosphorylation, or incorporation into other precursors which is presumably low but not quantified. The kit used is designed to assay 2DG6P, which is only intracellular, so we can't see the release into the medium. 2DG could be assayed in the medium but its presumed low concentration after export should be vastly outcompeted by glucose. In short, we see no easy (and non-radioactive) way of quantifying this. In the Results section, we only mentioned that “*the endocytosis blockade caused by the deletion of ROD1 allows a better detoxification of 2DG*” so we left this statement. In the discussion, we toned down as follows.

We added the following statement :

*“Thus, stabilizing glucose transporters at the plasma membrane might allow the release of 2DG back to the medium, but this possibility should be further explored in the future”*
and removed the following ;

*“Altogether, we propose that 2DG-induced endocytosis is detrimental to cells, and that deletion of ROD1 partially corrects this by stabilizing glucose transporters, leading to a sustained glycolysis and 2DG export.”*

We also added a question mark to the model in Figure 7.

See also response to point 5, Reviewer 2.

10. Fig. 6E: The FRET experiments indicate a slightly more glucose uptake in rod1 mutants after 4h of 2DG treatment and subsequent glucose addition. I suggest that the authors use the same approach to compare glucose influx in WT cells and rod1 mutants during the 4h 2DG treatment (provided of course the sensor is not activated by 2DG).
Thank you for the interesting suggestion. This could be done in principle as 2DG only binds the Glc sensor with a weak affinity and so it does not interfere with the measurement (Fehr et al. 2003). This important information was omitted from our manuscript and is now added in the text.

The experiment proposed by the Reviewer is exactly what we initially had in mind (measure glucose accumulation over time in WT vs *rod1∆* after 2DG treatment) but we faced several technical problems. First, the growth medium interferes significantly with the measurement, and indeed these experiments were initially set up in the original paper and in our lab with cells resuspended in MES buffer pH 6.0 (Bermejo et al. 2010 PMID 20854260). Second, *rod1∆* grows in the presence of 2DG whereas the WT does not, which leads to artifactual differences over time. Finally, this measurement would tell us how much free glucose is inside the cells at equilibrium, rather than measuring "influx". Influx is better approximated by washing the cells and measuring the variation of intracellular glucose levels upon glucose addition, as shown in Fig. 6.

**Reviewer #2:**

In the manuscript by Laussel et. al entitled, "2-deoxyglucose inhibits yeast AMPK signaling and triggers glucose transporter endocytosis, potentiating the drug toxicity", the authors investigated toxicity/resistance mechanisms of 2-deoxyglucose (2DG) in the model organism Saccharomyces cerevisiae. This is potentially an important piece of work that should be of interest to a broad audience as 2DG and its derivatives have anticancer potential and are used in cancer diagnostics.
Common resistance mechanism to 2DG involves constitutive activation of the 5’-AMP activated protein kinase (AMPK) Snf1 by mutating GLC7 and REG1 genes encoding subunits of the Protein Phosphatase 1 (PP1) complex, a negative regulator of Snf1. This leads to increased expression of DOG1 and DOG2 that encode phosphatases catalyzing dephosphorylation of 2DG-6-phosphate, a toxic metabolite of 2DG. It has been also shown that 2DG triggers alpha-arrestin (Rod1 and Rog3)-dependent endocytic degradation of Hxt1 and Hxt3 glucose transporters (O’Donnell et al., 2015, MCB). In addition, it was observed that 2DG treatment leads to increased phosphorylation and ubiquitination of Rod1 that requires Snf1 and PP1, respectively. This is consistent with the Snf1 role in preventing ubiquitination and activation of Rod1 when glucose is scarce. It was proposed that either 2DG increases activity of Snf1 leading to hyperphoshorylation and ubiquitination of Rod1 or Snf1-dependent phosphorylation of Rod1 is always inhibitory but 2DG simultaneously increases activity of PP1 towards Rod1 and/or Snf1. In sum, O’Donnell et al. proposed that 2DG toxicity largely results from glucose uptake inhibition caused by 2DG-induced internalization of glucose transporters.
In this work, the group of Sébastien Léon investigated the role of Snf1/PP1 pathway in Rod1-dependent internalization of glucose transporters Hxt1 and Hxt3 during 2DG treatment in more detail. The major novel findings are:
• 2DG addition triggers immediate but transitory dephosphorylation and inhibition of Snf1 that depends on PP1;
• 2DG treatment also induces rapid dephosphorylation of other Snf1 targets, such as Rod1 and Mig1, strongly suggesting upregulation of PP1 activity in the presence of 2DG;
• Phosphorylation of 2DG to 2DG-6-P by Hxk2 is required to promote PP1-independent dephosphorylation;
• 2DG toxicity largely results from Rod1-dependent internalization of glucose transporters and thus impairing both glucose uptake and detoxification of 2DG;
• 2DG addition triggers internalization of several plasma membrane proteins, also independently from Rod1.

Unfortunately, in my opinion, many conclusions are not convincingly supported by the presented data.

Thank you for the careful examination and the frank assessment of our manuscript.

Major points:
1. The authors showed that 2DG treatment cause internalization of several plasma membrane proteins in both Rod1-dependent and independent way, including previously reported Hxt1 and Hxt3 (Figs 1, S2, S3, S5) . This is an interesting observations but this set of data is somehow detached from the rest of manuscript.
We understand that this part may appear dissociated from the rest of the manuscript. Chronologically, we started investigating the global endocytosis response to 2DG but soon realized that Rod1 was critical in this response, confirming findings by O'Donnell et al. 2015. We came back to this story when we realized that 2DG transiently inhibited Snf1, thinking that this may (1) provide clues as to why Rod1 is "activated" in these conditions and thus may explain the sudden endocytosis response and (2) provide crucial information as to how 2DG works differently in yeast as it does in mammalian cells.
It is not clear whether internalization of these plasma membrane proteins, except for Hxt1 and Hxt3, also contributes to 2DG toxicity. We toned down our argument at the closing of the introduction by changing “*Finally, we demonstrate that 2DG resistance of the rod1∆ strain can solely be attributed to the maintenance of active glucose transporters at the membrane*” into “*Finally, we demonstrate that 2DG resistance of the rod1∆ strain relies on the maintenance of active glucose transporters at the membrane*”.

Regarding how *rod1*∆ becomes resistant to 2DG, we expected that one or more Rod1 cargoes would contribute so we first tried with Hxt1 and Hxt3, as presented in our manuscript. We believe that the genetic interactions we report (*rod1∆* vs *rod1∆ hxt∆)* provides strong support to the previously published hypothesis that lack of glucose transporter endocytosis contributes to 2DG resistance. We did not systematically address whether the deletion of other transporters would also restore the 2DG sensitivity of a *rod1∆* mutant, however we show that the deletion of *HXT6* has no impact (Fig 5C) suggesting some level of specificity, even in the family of glucose transporters.
For Rod1-independent endocytosis, the authors could test involvement of other alpha-arrestins that might be also modified in response to 2DG by PP1-dependent dephosphorylation or other mechanisms. These data would be a good starting point for a separate story and should be deleted from the current manuscript.

Concerning the Rod1-independent, 2DG-induced endocytosis of cargoes, we are not aware of another arrestin that would be regulated in these conditions, except for the obvious Rog3/Art7 which we found not to be involved in the endocytosis of the Rod1-independent cargo Lyp1(Fig S3). The quest for the arrestin involved, and then making sense of its regulation can be a tedious task and a long process, particularly in the context of the now well-described condition of 2DG-induced endocytosis. So we'd rather keep the manuscript as it is, especially since other reviewers did not raise comments about this.

2. One of major conclusions is that 2DG-6-P increases activity of PP1. However, this assumption is solely based on decrease in phosphorylation levels of PP1 targets such as Snf1 and Mig1. Such conclusion should be supported by direct measurements of PP1 enzymatic activity after addition of 2DG and/or testing increased physical interactions of PP1 with its substrates as an alternative hypothesis. In my opinion, presented results only support the notion that 2DG-6-P promotes/facililates PP1-dependent dephosphorylation of its targets by unknown mechanism.
Thank you for this comment which encouraged us to think more deeply about it.

We agree that we miss a direct measurement of PP1 activity to be able to state that 2DG increases activity of PP1. This is a critical aspect of 2DG signaling that we plan to further study. Until then, we toned down our conclusions related to PP1 "activation" by 2DG and restricted our conclusions to its involvement in 2DG-induced dephosphorylation of Snf1 and its substrates..

- Abstract :
  - ”*Mechanistically, this is explained by a transient, 2DG-induced activation of protein phosphatase 1 (PP1) and a concomitant inactivation of Snf1/AMPK*. ”
  - becomes: “*Mechanistically, this is explained by a transient, 2DG-induced inactivation of Snf1/AMPK by protein phosphatase 1 (PP1).*”
- Introduction, l. 128 :
  - “*Mechanistically, we show that opposite to the situation in mammalian cells, 2DG treatment leads to the dephosphorylation of Snf1/AMPK and several targets including Rod1, likely through a transient surge in PP1 activity.* ”
  - becomes: *“Mechanistically, we show that opposite to the situation in mammalian cells, 2DG treatment leads to the dephosphorylation of Snf1/AMPK and several targets including Rod1 in a-PP1 dependent manner”*
- Results, l.207
  - “*The fact that 2DG triggers Rod1 dephosphorylation led us to think that this may occur through a transient activation of PP1. Instead, 2DG was previously described to induce a mild phosphorylation (activation) of the PP1 substrate Snf1 within 2h after treatment with 2DG [16], similar to observations in mammalian cells [3, 39].*”
  - becomes: “*The fact that 2DG triggers a PP1-dependent dephosphorylation of Rod1 contrasted with previous results showing that 2DG induces a mild phosphorylation (activation) of the PP1 substrate Snf1 within 2h of treatment [16], similar to observations in mammalian cells [3, 39].* ”
- Results, line 253:
  - “*Altogether, we propose that 2DG phosphorylation by Hxk2 triggers a transient activation of PP1, leading to Snf1 dephosphorylation, Rod1 dephosphorylation and endocytosis.*”
  - becomes: “*Altogether, we propose that 2DG phosphorylation by Hxk2 triggers a PP1-dependent Snf1 dephosphorylation, Rod1 dephosphorylation and endocytosis.*”
- Discussion, line :
  - we deleted “*a sudden increase in PP1 activity in response to 2DG would also account for the changes in Rod1 post-translational modifications*”
  - and discussed potential mechanisms by which Snf1 substrate dephosphorylation may occur (also in response to point 10): l. 374-389
- Discussion, line 402-403:
  - “.*.. suggesting that 2DG6P (or a derived metabolite) contributes to the potential surge of PP1 activity.* ”
  - becomes: *“suggesting that 2DG6P (or a derived metabolite) contributes to the dephosphorylation of Snf1 and its substrates”.*

We believe that these statements are more rigorous and so we would like to thank the referee again for the suggestion.

3. Changes in phosphorylation status of Rod1 after 2DG treatment for 10 min are not convincing due to overlap of bands corresponding to phosphorylated and ubiquitinated forms of Rod1 (Fig. 2A-D). I do not notice any significant change in the intensity of upper band after CIP treatment in WT + 2DG, 10’ compared to –CIP sample (Fig. 2B). It is also difficult to judge changes in phosphorylation status of Rod1-KR devoid of ubiquitination site (Fig 2A).
We think that there is a misunderstanding here. We fully agree with the reviewer that there is no difference in the upper (ubiquitylated) bands before and after CIP treatment, which to us indicates that contrary to what was suggested before, Rod1 is not phosphorylated in response to 2DG.

When the "overlap" between Phospho-Rod1 and Ubi-Rod1 is removed by using the non-ubiquitylatable Rod1-KR mutant (Fig 2A), there is also not much difference before/after 2DG treatment. Overall, this is consistent with our model that shortly after treatment, Snf1 is inhibited rather than activated, and Rod1 is dephosphorylated and ubiquitylated rather than being phosphorylated. So overall, our conclusions are in line with the reviewer’s conclusions.

4. It would be very informative to follow post-translational modifications of Rod1 and Mig1 as well as localization of Hxt1-GFP or Hxt3-GFP after 2DG addition at several time-points as it was done for Snf1 phosphorylation in Fig. 3C.
We had already looked at Mig1 (panel 3D) and we now provide an extended timecourse for Rod1 post-translational modifications and Snf1 phosphorylation in Fig 3C (see also Reviewer 1, point 6). The data confirms the pattern observed for Snf1 phosphorylation, i.e. a strong dephosphorylation in response to 2DG at early time points, followed by rephosphorylation later on.

Concerning the localization of Hxt1-GFP and Hxt3-GFP at several time-points, we have looked at Hxt3 (below) but we think it does not bring additional information so we would rather keep the data on endocytosis as it is.

5. Based on data shown in Fig. 6A the authors concluded that deletion of ROD1 (and stabilization of hexose transporters) contributes to 2DG detoxification. However, it is not clear which substrate is detoxify, 2DG or 2DG-6-P? Similarly in the text, both forms are mentioned as subjected to detoxification (lines 305-306). Involvement of Hxt1 or Hxt3 in the detoxification process of 2DG is not demonstrated. In rod1Δ cells, efflux of 2DG or 2DG-6-P might be facilitated by other transporters stabilized in the plasma membrane. In general, more elaborated transport studies are needed to prove detoxification of 2DG/2DG-6-P and its mechanism.
Sorry for the lack of clarity. We used "detoxification" for any process leading to the lack of toxicity, including dephosphorylation of 2DG6P or potential export of the resulting 2DG (since glucose transporters only transport non-phosphorylated sugars). This has been clarified in the text (l. 113: “*detoxicate”* changed to “*dephosphorylate”*).

We agree that a potential 2DG efflux could be mediated by other transporters, however genetic evidence indicates that Hxt1 and Hxt3 are important players for 2DG toxicity, with a prominent role of Hxt3 (see Fig 5D). To test our assumption that Hxt3 is involved in 2DG release, we performed the same chase experiment as presented in Panel 6B but now we checked the involvement of Hxt3 in 2DG6P decrease. This is now presented in panel 6D. First, the results confirm those obtained in panel 6B, i.e. there is a much lower amount of 2DG6P in *rod1∆* cells than in WT cells after 30’ of chase. Second, they also reveal that the additional deletion of *HXT3* in the *rod1∆* mutant reverts 2DG6P levels back to those of the WT, suggesting that Hxt3 takes part in 2DG6P detoxification. Of note, there were no significant differences between the WT and the single *hxt3∆* mutant. A simple explanation is that *rod1∆* cells show a decreased 2DG6P content because the maintenance of Hxt3 at the cell surface favors 2DG exit after its dephosphorylation, thereby preventing another round of phosphorylation.

However, we agree that more elaborate transport studies would be required to be fully conclusive. We toned down our discussion by adding the following in the discussion : *“Thus, stabilizing glucose transporters at the plasma membrane might allow the release of 2DG back to the medium, but this possibility should be further explored in the future”.*

See also response to Reviewer 1, point 9.

Other comments:
1. Lines 141-142: “0.2% 2DG led to the degradation of PM-localized Tat1 and Hxt2 (Fig 1A), which was accompanied by their targeting to the vacuole (Fig 1B).”; it is better to say that “PM-localized Tat1 and Hxt2 were internalized and sorted to the vacuole for degradation. Panels Fig. 1A and 1B should be swapped.
We followed the reviewer's suggestions, thank you.

2. Line 151: “2DG triggers a general endocytosis response”. This is unclear statement and not true as the complete plasma membrane proteome was not tested to conclude this.
We were looking for a term that recapitulates our observation that all transporters (13/13) we studied are indeed endocytosed in this condition. But we agree that this is not accurate and we removed the comment.

3. Lines 158-159: “Altogether, these results indicate a prominent role for the ART protein Rod1 in 2DG-induced endocytosis.” This statement is too general as 2DG-induced internalization of many plasma membrane proteins are not dependent on Rod1. One could conclude that Rod1 is involved in 2DG-induced endocytosis of its targets.
Actually, only 3/12 are not Rod1-dependent so we believe we can claim that Rod1 is the prominent arrestin for transporters in these conditions.

4. Fig. 2A-B: please label bands of Rod1 as unmodified and Ub/P forms.
We followed the reviewer's suggestions, thank you.

5. Fig. 2E-G and Fig. 4E-F: it is unclear to me why Ina1-GFP was used as a model cargo instead of Hxt1 or Hxt3, which are central to this paper.
We wanted to stay away from naturally glucose-regulated cargoes (see l. 194). However, and following Reviewer 1’s comments, there are now a number of experiments which have also been repeated for Hxt1 and Hxt3.

6. Fig. 3A: Total proteins label is missing.
Corrected, thank you

7. In Fig. 5D, the rod1Δ mutant with empty plasmid shows poor growth in the presence of 0.2% 2DG, whereas this strain shows a robust growth on 0.2% 2DG plates in other panels (Figs 2F, 5C-D).
This is now Fig 5C. Indeed, the *rod1∆* strain does not grow as well as other strains on this plate (eg. *rod1∆* overexpressing *HXT3*) but it still grows better than WT cells. We now provide the same plate after 4d of growth instead of 3d to make this more evident.

8. Line 247: “ROD1 deletion leads to a partial 2DG resistance [14]”; How partial, compare to what? Please, specify.
*ROD1* deletion confers a weaker resistance than *REG1* deletion or the double *ROD1* and *ROG3* deletion, for instance. Please note that this is mentioned elsewhere in the text (l. 117). But at this stage in the paper, this information is not essential so we removed “a partial“ from this sentence.

9. Lines 257-258: ”Thus, it seems that only a partial endocytosis defect such as that observed in the rod1Δ (or rod1Δ rog3Δ) mutant causes 2DG resistance”. What is a partial endocytosis defect? This is rather a defect in endocytosis of certain proteins targeted by Rod1 or both Rog3 and Rod1.
Sorry for the lack of clarity. By "partial endocytosis defect" we meant that *ROD1* deletion only partially blocks transporter endocytosis since a few cargoes are still internalized (Fig S2) and this equilibrium between cargoes that are stabilized whereas others are endocytosed may contribute to 2DG resistance. However, we think that our conclusion based on the lack of 2DG resistance of the *rvs167∆* mutant does not allow us to conclude on the relationship between 2DG and endocytosis, we have now removed this comment (also see response to reviewer 1, point 7).

10. Discussion lacks information about known mechanisms of PP1 regulation.
The fact that Snf1 is mostly regulated at the level of its dephosphorylation by PP1 has clearly appeared (Rubenstein et al. 2008, PMID 17991748), however much less is known on how PP1 activity in response to glucose is regulated. In the Discussion section, we now summarize the existing knowledge and suggest potential mechanisms by which 2DG may regulate AMPK activity. (see paragraph starting at line 374).

11. Page 17: The authors describe the method of Ina1-GFP purification for ubiquitylation analysis but there are no such data presented in this paper.
Apologies, this corresponded to a previous version of the manuscript..

12. Figure 6 legend, line 956: 0,2% DG instead of 0.2% 2DG; line 967: 0,1% DG instead of 0.1% 2DG; why 0.1% 2DG was used instead of 0.2% in panel Fig. 6F?
Thank you, this is now corrected. As for the panel 6F, we simply used the 0.1% 2DG plate because the 0.2% 2DG plate was contaminated, but this does not change our conclusions.

13. Figure 5 and 6 legends: please clearly state for how many days the plates were incubated instead of saying 3-5 days.
Corrected, thank you.

**Reviewer #3:**

The paper entitled “2-deoxyglucose inhibits yeast AMPK signaling and triggers glucose transporter endocytosis, potentiating the drug toxicity” reports that 2DG regulates the endocytosis of many plasma membrane proteins, mostly in a Rod1-dependent manner. The data reported in this paper are interesting and they fit into a logical model. The quality of the work is high, indicating that the experiments were carried out with great care and precision. However, a number of results are rather confirmatory of previously published data (Refs. 16, 28, 31, 32).
Thank you for taking the time to read and evaluate our work. We agree that some results are confirmatory, but also believe that many of our results shed a new light on previous results as well as new effects of 2DG on signaling.

Majors:
1. Because addition of glucose or 2DG to glucose-starved cells results in a sudden increase in PP1 activity, the authors state that: “*Snf1 dephosphorylation by 2DG required the PP1 subunit Reg1, thus mimicking the effect that glucose has on glucose-starved cells* (lines 213-214).” and that: “*this mimicked the situation described when glucose-starved cells are treated with glucose* (lines 217-218).” However, these statements are premature and lack adequate experimental evidence. Given that Rod1 is activated (ubiquitinated) in response to high glucose, the low affinity glucose transporters Hxt1 and Hxt3 would be primary targets of Rod1 (according to their claim) under such conditions. However, they are not endocytosed but are stable and remain functional when cells are exposed to high glucose levels. Although PP1 activity is increased in response to both high glucose and 2DG, the glucose transporters are endocytosed in response to 2DG but not to high glucose. The authors should better differentiate between high glucose-induced endocytosis (Jen1) and 2DG-induced endocytosis (Hxt1 and Hxt3) in glucose- grown cells.
We think we understand the apparent paradox that the reviewer is referring to, and we think this is caused by a lack of clarity in our manuscript.
- Yeast *growing on high glucose* are adapted to high glucose - and despite the presence of high glucose, Snf1 is mildly active: it is partially phosphorylated, and so are its substrates (Fig 2: "Glc" conditions for Rod1, and Fig 4 for Snf1 and Mig1). Thus, Rod1 is not ubiquitylated in high-glucose grown cells, and this corresponds to a state in which endocytosis of Hxt1-Hxt3 etc. does not occur.
- Instead, when glucose is *added after glucose starvation*, Snf1 (which was strongly active before the shift) is abruptly inactivated through PP1-mediated dephosphorylation and this condition causes Rod1 activation.
This is why we are stating that 2DG acts *like glucose does on glucose-starved cells* - i.e. a glucose "recovery" situation despite the fact that cells were previously on high glucose medium. We think that 2DG causes an aberrant activation of Rod1, leading to increased endocytosis.

2. 2DG-induced phosphorylation/activation of Snf1 is modest yet sufficient for the increased phosphorylation of Rod1 (Ref. 16).

As discussed above (Reviewer 2, point 3), we actually have no evidence that Rod1 is phosphorylated in response to 2DG. Rather, the sudden change in its post-translational modifications (ubiquitylation) indicates that it is instead dephosphorylated, since the same pattern is obtained for a non-phosphorylatable Rod1 (Fig 2D)

The authors observed a sharp but transient decrease in Snf1 phosphorylation shortly after 2DG addition. Their results show that Snf1 activity decreases immediately after 2DG treatment and increases within 1⁄2 - 1 hr after 2DG treatment (Figs. 3 B and 3C). However, the investigation of the functional significance of the 2DG-induced transient inactivation of Snf1 was not properly conducted. 2DG-induced endocytosis was not assessed by fluorescence microscopy until 4 hours after 2DG treatment. One can assume that Snf1 is active in cells treated with 2DG for more than an hour and regulates 2DG-indcued endocytosis.

We agree that the timings for the "signaling" experiments differ from those of the "endocytosis" experiments. Although the effect of 2DG on Rod1 activation is almost immediate, membrane trafficking is a much slower process, particularly in the case of energy-deprived cells like 2DG treated cells. Similar results were obtained on other arrestins in starvation conditions, whereby starvation has an almost immediate effect on arrestin regulation but takes hours to translate into endocytosis (Ivashov et al. 2021, Hovsepian et al. 2017). Like in these cases, Rod1 may act only at the early steps of endocytosis (at the plasma membrane) but endocytosis is better visualized after internalization, endosomal trafficking, and vacuolar delivery which can overall take up hours to complete. Finally, our new results presented in panel 3C now indicate that Rod1 remains ubiquitylated and thus presumably active long after 2DG treatment.

3. The authors claim that Rod1-flag behaves functionally as the wild-type protein, because expression of Rod1-3HA does not restore endocytosis nor sensitivity to 2DG in a *rod1Δ* context, contrary to Rod1-flag (Fig. S4). Interestingly, the *rod1Δ* mutant is shown to be resistant to 2DG in figures 5B, 5C, and 6F but not in Fig. S4B upper panel, which might cause misinterpretation of the results. This should be clarified to avoid any confusion in the matter.
Thank you for pointing out these apparent discrepancies and allowing us to correct these points. Panel S4B was remade as per Reviewer 1's request (comment 1).
However, expression of Rod1-3HA restores sensitivity (Fig. S4B) and endocytosis of Hxt1 (Ref. 32) and Jen1 (Ref. 28) in a *rod1*Δ context.
To the best of our knowledge, Ref 32 used untagged Rod1 in the complementation experiments (and accessorily, it was expressed from high-copy 2µ vectors). Our data unambiguously show that Rod1-HA is not competent for Hxt1 or Hxt3 endocytosis (fig S4A). The fact that Rod1-3HA is functional for Jen1 endocytosis in response to glucose is indeed mentioned in the text (l. 172) and was published by us, which explains why we had started this study with Rod1-3HA as well. We can only conclude that there may be slight differences between 2DG- and glucose-induced endocytosis, since a tag may affect only a subset of the function or regulation of a given protein in a given context.

4. The lack of quantification of the plasma membrane and intracellular GFP fluorescence intensities measured in this manuscript does not allow a rigorous evaluation of protein turnover and recruitment. For example, vacuolar fragmentation is observed in 2DG-treated cells, but it is unclear whether Ina1-GFP is endocytosed in response to 2DG. 2DG (or some drugs) causes ER stress-induced vacuolar fragmentation (Fig. S1), which however does not directly reflect protein endocytosis.
Although we would like to stay away from Ina1 as much as possible to avoid diluting the main message of our study (see Minor point 1, below), we now provide results confirming that Ina1-GFP is endocytosed in response to 2DG: Ina1-GFP co-localizes with the vacuolar dye CMAC after 2DG treatment, and this vacuolar localization requires the endocytosis protein Rvs167 (new Fig S5A). This is now added in the text.

The results would have also been stronger if immunoblot analysis (of plasma membrane fractions) were provided to back up the fluorescence microscopy.
We now provide immunoblot analysis (Fig S5B) showing that Ina1-GFP is degraded in response to 2DG, concomitant to the appearance of free GFP moiety indicative of vacuolar degradation (as shown for Hxt2 and Tat1, Fig 1). This is complicated by the fact that Ina1 is a heavily glycosylated protein but we treated the samples with endoglucanase H to clean up the migration profile. This is now added in the text.

5. The abstract of the paper claims that: “Rod1 is central to 2DG-induced endocytosis because 2DG, following its phosphorylation by hexokinase Hxk2, triggers changes in Rod1 post-translational modifications and promotes its function in endocytosis.” However, there are many PM proteins that are endocytosed in a 2DG- induced, Rod1-independent manner (Fig. S2). Furthermore, expression of a constitutively active (dephosphorylated/ubiquitinated) Rod1 (Rod1-S12A-Flag) does not lead to 2DG- independent endocytosis of Ina1-GFP (Fig. 2), suggesting that 2DG might have a limited role in translational modification of Rod1 (phosphorylation & ubiquitination). This sentence should be toned down.
As far as we can tell, there are only 3/13 proteins that are endocytosed independently of Rod1. However, we fully agree that there must be additional mechanisms contributing to triggering endocytosis in addition to those acting on Rod1, as stated in our manuscript (l.202-203). We toned down this sentence and instead state that Rod1 “participates in” 2DG-induced endocytosis.

Minors:

1. The title of the manuscript does not reflect its contents accurately. It is unclear whether 2DG inactivation of Snf1 is required for endocytosis, because the effect on Snf1 activity is transitory.

Agreed, but yet endocytosis is affected

-upon deleting *ROD1*,

-when preventing Rod1 ubiquitylation (Rod1-KR mutant),

-and this ubiquitylation requires PP1 activation or at least Snf1 inactivation to happen

so we believe that this initial response (Snf1 inactivation) to 2DG is required to trigger endocytosis.

Also, we show that Snf1 activity is impacted for longer than the 10 min we had looked at initially (see new panel 3C).

In addition, the main focus of the paper is actually on Ina1 but not on glucose transporters. This should be reflected in the title.
Ina1 is used as a proxy to measure endocytosis of a glucose-unrelated cargo but we have no functional data on its possible involvement in the process. On the contrary, a strong aspect of our study is to provide more insights into how 2DG affects signaling and how abrogating glucose transporter endocytosis impacts on 2DG resistance. Moreover, we now provide a collection of additional results on Hxt1 and Hxt3 which back up our results on Ina1. So we kindly ask to keep the title as it is.

2. The authors also investigated how *ROD1* deletion results in 2DG resistance and concluded that stabilization of Hxt1 and Hxt3 glucose transporters confers 2DG resistance to the *rod1*Δ mutant. These results are what would be expected, given that Rod1 removal increases the growth of 2DG-treated cells (Ref. 16, 32).
Indeed, *ROD1* deletion causes resistance to 2DG and this correlates with stabilization of glucose transporters. But our data now show that removal of *HXT1* or *HXT3* in the context of the *rod1∆* strain suppresses this resistance, proving causality between these two events.
